# Supplementary material for: A duo-theme cloud model DEMATEL approach for exploring the cause factors of green supply chain management
Source: PLoS One. 2024 Mar 28;19(3):e0294684. doi: 10.1371/journal.pone.0294684 (PMC10977737; doi:10.1371/journal.pone.0294684)
Supplement: S1 Appendix — (DOCX) [file pone.0294684.s001.docx]

Appendix

I**.** Three key parameters of the direct-relation matrix

| Ce | S1 | S2 | S3 | S4 | S5 | S6 | S7 | S8 | S9 | S10 | S11 | S12 |
| --- | --- | --- | --- | --- | --- | --- | --- | --- | --- | --- | --- | --- |
| S1 | (0.326,0.172,0.148) | (9.533,0.247,0.183) | (0.433,0.862,0.223) | (3.185,0.898,0.612) | (2.154,0.792,0.387) | (1.666,0.983,0.765) | (3.185,0.898,0.612) | (2.154,0.792,0.387) | (1.666,0.983,0.765) | (1.363,0.963,0.237) | (1.312,0.838,0.347) | (1.312,0.838,0.347) |
| S2 | (1.363,0.963,0.237) | (0.433,0.862,0.223) | (3.185,0.898,0.612) | (2.896,0.476,0.428) | (1.363,0.963,0.237) | (1.312,0.838,0.347) | (0.326,0.172,0.148) | (0.433,0.862,0.223) | (0.326,0.172,0.148) | (1.888,0.782,0.632) | (1.888,0.782,0.632) | (1.888,0.782,0.632) |
| S3 | (1.312,0.838,0.347) | (0.326,0.172,0.148) | (9.533,0.247,0.183) | (3.185,0.898,0.612) | (1.312,0.838,0.347) | (1.312,0.838,0.347) | (0.433,0.862,0.223) | (0.278,0.693,0.127) | (0.326,0.172,0.148) | (1.312,0.838,0.347) | (2.154,0.792,0.387) | (1.363,0.963,0.237) |
| S4 | (3.185,0.898,0.612) | (0.333,0.193,0/178) | (0.333,0.193,0/178) | (0.326,0.172,0.148) | (1.312,0.838,0.347) | (2.154,0.792,0.387) | (1.312,0.838,0.347) | (1.363,0.963,0.237) | (1.888,0.782,0.632) | (1.312,0.838,0.347) | (1.666,0.983,0.765) | (1.312,0.838,0.347) |
| S5 | (9.533,0.247,0.183) | (0.433,0.862,0.223) | (9.533,0.247,0.183) | (1.312,0.838,0.347) | (0.433,0.862,0.223) | (2.896,0.476,0.428) | (1.363,0.963,0.237) | (1.888,0.782,0.632) | (1.312,0.838,0.347) | (3.185,0.898,0.612) | (2.896,0.476,0.428) | (2.154,0.792,0.387) |
| S6 | (1.312,0.838,0.347) | (0.326,0.172,0.148) | (0.333,0.193,0/178) | (0.433,0.862,0.223) | (1.888,0.782,0.632) | (0.326,0.172,0.148) | (0.333,0.193,0/178) | (1.312,0.838,0.347) | (2.896,0.476,0.428) | (2.896,0.476,0.428) | (1.888,0.782,0.632) | (1.363,0.963,0.237) |
| S7 | (1.888,0.782,0.632) | (1.363,0.963,0.237) | (1.363,0.963,0.237) | (1.888,0.782,0.632) | (1.363,0.963,0.237) | (1.312,0.838,0.347) | (0.326,0.172,0.148) | (2.896,0.476,0.428) | (3.185,0.898,0.612) | (1.312,0.838,0.347) | (3.185,0.898,0.612) | (1.666,0.983,0.765) |
| S8 | (1.363,0.963,0.237) | (9.533,0.247,0.183) | (2.154,0.792,0.387) | (2.154,0.792,0.387) | (1.666,0.983,0.765) | (1.888,0.782,0.632) | (0.333,0.193,0/178) | (9.533,0.247,0.183) | (0.433,0.862,0.223) | (0.433,0.862,0.223) | (2.896,0.476,0.428) | (1.888,0.782,0.632) |
| S9 | (2.896,0.476,0.428) | (0.326,0.172,0.148) | (0.433,0.862,0.223) | (1.363,0.963,0.237) | (1.312,0.838,0.347) | (1.312,0.838,0.347) | (9.533,0.247,0.183) | (1.666,0.983,0.765) | (0.512,0.483,0.227) | (1.363,0.963,0.237) | (1.888,0.782,0.632) | (1.363,0.963,0.237) |
| S10 | (0.326,0.172,0.148) | (1.312,0.838,0.347) | (0.326,0.172,0.148) | (9.533,0.247,0.183) | (1.312,0.838,0.347) | (1.363,0.963,0.237) | (0.433,0.862,0.223) | (0.326,0.172,0.148) | (9.533,0.247,0.183) | (0.326,0.172,0.148) | (1.363,0.963,0.237) | (1.312,0.838,0.347) |
| S11 | (2.154,0.792,0.387) | (0.333,0.193,0/178) | (2.896,0.476,0.428) | (1.888,0.782,0.632) | (1.888,0.782,0.632) | (1.363,0.963,0.237) | (9.533,0.247,0.183) | (1.888,0.782,0.632) | (1.312,0.838,0.347) | (2.896,0.476,0.428) | (0.326,0.172,0.148) | (1.312,0.838,0.347) |
| S12 | (1.312,0.838,0.347) | (1.363,0.963,0.237) | (3.185,0.898,0.612) | (3.185,0.898,0.612) | (1.363,0.963,0.237) | (1.363,0.963,0.237) | (0.326,0.172,0.148) | (1.312,0.838,0.347) | (0.433,0.862,0.223) | (2.154,0.792,0.387) | (3.185,0.898,0.612) | (0.326,0.172,0.148) |
|  |  |  |  |  |  |  |  |  |  |  |  |  |
| Cg | G1 | G2 | G3 | G4 | G5 | G6 | G7 | G8 | G9 | G10 |  |  |
| G1 | (0.326,0.172,0.148) | (2.154,0.792,0.387) | (1.666,0.983,0.765) | (0.433,0.862,0.223) | (1.312,0.838,0.347) | (1.363,0.963,0.237) | (0.326,0.172,0.148) | (3.185,0.898,0.612) | (1.888,0.782,0.632) | (1.312,0.838,0.347) |  |  |
| G2 | (2.154,0.792,0.387) | (9.533,0.247,0.183) | (1.888,0.782,0.632) | (1.312,0.838,0.347) | (1.363,0.963,0.237) | (1.312,0.838,0.347) | (1.363,0.963,0.237) | (2.896,0.476,0.428) | (2.896,0.476,0.428) | (2.896,0.476,0.428) |  |  |
| G3 | (3.185,0.898,0.612) | (1.363,0.963,0.237) | (0.326,0.172,0.148) | (0.333,0.193,0/178) | (3.185,0.898,0.612) | (2.896,0.476,0.428) | (1.312,0.838,0.347) | (0.433,0.862,0.223) | (1.363,0.963,0.237) | (3.185,0.898,0.612) |  |  |
| G4 | (0.433,0.862,0.223) | (1.888,0.782,0.632) | (0.433,0.862,0.223) | (0.326,0.172,0.148) | (1.312,0.838,0.347) | (1.312,0.838,0.347) | (0.433,0.862,0.223) | (9.533,0.247,0.183) | (1.312,0.838,0.347) | (1.312,0.838,0.347) |  |  |
| G5 | (2.896,0.476,0.428) | (2.154,0.792,0.387) | (2.154,0.792,0.387) | (1.888,0.782,0.632) | (0.326,0.172,0.148) | (1.888,0.782,0.632) | (1.888,0.782,0.632) | (2.154,0.792,0.387) | (1.666,0.983,0.765) | (1.888,0.782,0.632) |  |  |
| G6 | (3.185,0.898,0.612) | (1.666,0.983,0.765) | (3.185,0.898,0.612) | (1.363,0.963,0.237) | (2.896,0.476,0.428) | (0.326,0.172,0.148) | (2.154,0.792,0.387) | (1.888,0.782,0.632) | (1.888,0.782,0.632) | (2.154,0.792,0.387) |  |  |
| G7 | (1.312,0.838,0.347) | (1.888,0.782,0.632) | (1.312,0.838,0.347) | (0.333,0.193,0/178) | (0.433,0.862,0.223) | (9.533,0.247,0.183) | (9.533,0.247,0.183) | (0.433,0.862,0.223) | (3.185,0.898,0.612) | (2.896,0.476,0.428) |  |  |
| G8 | (1.888,0.782,0.632) | (3.185,0.898,0.612) | (9.533,0.247,0.183) | (9.533,0.247,0.183) | (1.363,0.963,0.237) | (1.363,0.963,0.237) | (1.888,0.782,0.632) | (0.326,0.172,0.148) | (1.312,0.838,0.347) | (1.888,0.782,0.632) |  |  |
| G9 | (0.433,0.862,0.223) | (2.896,0.476,0.428) | (1.363,0.963,0.237) | (0.433,0.862,0.223) | (3.185,0.898,0.612) | (1.666,0.983,0.765) | (1.312,0.838,0.347) | (1.363,0.963,0.237) | (0.433,0.862,0.223) | (1.363,0.963,0.237) |  |  |
| G10 | (1.363,0.963,0.237) | (3.185,0.898,0.612) | (1.312,0.838,0.347) | (0.326,0.172,0.148) | (1.312,0.838,0.347) | (1.312,0.838,0.347) | (2.154,0.792,0.387) | (1.363,0.963,0.237) | (1.312,0.838,0.347) | (0.326,0.172,0.148) |  |  |

II. The highest similarity corresponds to the closest assessment value

| Se | S1 | S2 | S3 | S4 | S5 | S6 | S7 | S8 | S9 | S10 | S11 | S12 |
| --- | --- | --- | --- | --- | --- | --- | --- | --- | --- | --- | --- | --- |
| S1 | 0 / 0.772 | 0 / 0.799 | 0 / 0.772 | 3 / 0.994 | 2 / 0.939 | 2 / 0.898 | 3 / 0.994 | 2 / 0.898 | 2 / 0.939 | 1 / 0.981 | 1 / 0.977 | 1 / 0.960 |
| S2 | 1 / 0.981 | 0 / 0.899 | 3 / 0.976 | 3 / 0.986 | 1 / 0.981 | 1 / 0.981 | 0 / 0.772 | 0 / 0.799 | 0 / 0.681 | 2 / 0.898 | 2 / 0.987 | 2 / 0.898 |
| S3 | 1 / 0.977 | 0 / 0.772 | 0 / 0.799 | 3 / 0.994 | 1 / 0.960 | 1 / 0.977 | 0 / 0.772 | 0 / 0.681 | 0 / 0.772 | 1 / 0.977 | 2 / 0.898 | 1 / 0.977 |
| S4 | 3 / 0.994 | 0 / 0.772 | 0 / 0.957 | 0 / 0.772 | 1 / 0.979 | 2 / 0.987 | 1 / 0.981 | 1 / 0.977 | 2 / 0.898 | 1 / 0.960 | 2 / 0.939 | 1 / 0.979 |
| S5 | 0 / 0.799 | 0 / 0.799 | 0 / 0.772 | 1 / 0.960 | 0 / 0.772 | 3 / 0.994 | 1 / 0.977 | 2 / 0.939 | 1 / 0.960 | 3 / 0.994 | 3 / 0.994 | 2 / 0.939 |
| S6 | 1 / 0.960 | 0 / 0.681 | 0 / 0.772 | 0 / 0.899 | 2 / 0.898 | 0 / 0.899 | 0 / 0.899 | 1 / 0.960 | 3 / 0.986 | 3 / 0.976 | 2 / 0.898 | 1 / 0.979 |
| S7 | 2 / 0.987 | 1 / 0.977 | 1 / 0.981 | 2 / 0.939 | 1 / 0.977 | 1 / 0.960 | 0 / 0.681 | 3 / 0.994 | 3 / 0.976 | 1 / 0.979 | 3 / 0.994 | 2 / 0.898 |
| S8 | 1 / 0.981 | 0 / 0.681 | 2 / 0.939 | 2 / 0.987 | 2 / 0.987 | 2 / 0.939 | 0 / 0.957 | 0 / 0.899 | 0 / 0.772 | 0 / 0.772 | 3 / 0.986 | 2 / 0.939 |
| S9 | 3 / 0.976 | 0 / 0.899 | 0 / 0.681 | 1 / 0.981 | 1 / 0.981 | 1 / 0.979 | 0 / 0.772 | 2 / 0.987 | 0 / 0.681 | 1 / 0.960 | 2 / 0.939 | 1 / 0.981 |
| S10 | 0 / 0.899 | 1 / 0.981 | 0 / 0.899 | 0 / 0.772 | 1 / 0.960 | 1 / 0.977 | 0 / 0.681 | 0 / 0.772 | 0 / 0.899 | 0 / 0.799 | 1 / 0.981 | 1 / 0.960 |
| S11 | 2 / 0.939 | 0 / 0.772 | 3 / 0.986 | 2 / 0.898 | 2 / 0.939 | 1 / 0.960 | 0 / 0.899 | 2 / 0.939 | 1 / 0.981 | 3 / 0.986 | 0 / 0.772 | 1 / 0.977 |
| S12 | 1 / 0.977 | 1 / 0.981 | 3 / 0.994 | 3 / 0.976 | 1 / 0.981 | 1 / 0.981 | 0 / 0.799 | 1 / 0.981 | 0 / 0.799 | 2 / 0.939 | 3 / 0.976 | 0 / 0.772 |
|  |  |  |  |  |  |  |  |  |  |  |  |  |
| Sg | G1 | G2 | G3 | G4 | G5 | G6 | G7 | G8 | G9 | G10 |  |  |
| G1 | 0 / 0.681 | 2 / 0.939 | 2 / 0.939 | 0 / 0.772 | 1 / 0.960 | 1 / 0.981 | 0 / 0.681 | 3 / 0.994 | 2 / 0.939 | 1 / 0.981 |  |  |
| G2 | 2 / 0.898 | 0 / 0.681 | 2 / 0.939 | 1 / 0.960 | 1 / 0.981 | 1 / 0.977 | 1 / 0.981 | 3 / 0.976 | 3 / 0.994 | 3 / 0.986 |  |  |
| G3 | 3 / 0.994 | 1 / 0.981 | 0 / 0.799 | 0 / 0.799 | 3 / 0.986 | 3 / 0.994 | 1 / 0.977 | 0 / 0.799 | 1 / 0.960 | 3 / 0.976 |  |  |
| G4 | 0 / 0.799 | 2 / 0.939 | 0 / 0.681 | 0 / 0.681 | 1 / 0.977 | 1 / 0.977 | 0 / 0.772 | 0 / 0.799 | 1 / 0.981 | 1 / 0.960 |  |  |
| G5 | 3 / 0.976 | 2 / 0.898 | 2 / 0.898 | 2 / 0.939 | 0 / 0.772 | 2 / 0.987 | 2 / 0.898 | 2 / 0.987 | 2 / 0.987 | 2 / 0.939 |  |  |
| G6 | 3 / 0.986 | 2 / 0.987 | 3 / 0.976 | 1 / 0.981 | 3 / 0.994 | 0 / 0.799 | 2 / 0.987 | 2 / 0.898 | 2 / 0.898 | 2 / 0.987 |  |  |
| G7 | 1 / 0.960 | 2 / 0.939 | 1 / 0.977 | 0 / 0.681 | 0 / 0.681 | 0 / 0.681 | 0 / 0.799 | 0 / 0.681 | 3 / 0.976 | 3 / 0.994 |  |  |
| G8 | 2 / 0.939 | 3 / 0.986 | 0 / 0.681 | 0 / 0.799 | 1 / 0.981 | 1 / 0.960 | 2 / 0.898 | 0 / 0.799 | 1 / 0.979 | 2 / 0.987 |  |  |
| G9 | 0 / 0.681 | 3 / 0.976 | 1 / 0.981 | 0 / 0.799 | 3 / 0.976 | 2 / 0.898 | 1 / 0.960 | 1 / 0.960 | 0 / 0.899 | 1 / 0.977 |  |  |
| G10 | 1 / 0.981 | 3 / 0.994 | 1 / 0.960 | 0 / 0.681 | 1 / 0.960 | 1 / 0.981 | 2 / 0.939 | 1 / 0.981 | 1 / 0.977 | 0 / 0.799 |  |  |

III. Direct-relation matrix of economy and greenness enablers

| Xe | SI | S2 | S3 | S4 | S5 | S6 | S7 | S9 | S10 | S11 | S12 |
| --- | --- | --- | --- | --- | --- | --- | --- | --- | --- | --- | --- |
| SI | 0 | 0 | 0 | 3 | 2 | 2 | 3 | 2 | 1 | 1 | 1 |
| S2 | 1 | 0 | 3 | 3 | 1 | 1 | 0 | 0 | 2 | 2 | 2 |
| S3 | 1 | 0 | 0 | 3 | 1 | 1 | 0 | 0 | 1 | 2 | 1 |
| S4 | 3 | 0 | 0 | 0 | 1 | 2 | 1 | 2 | 1 | 2 | 1 |
| S5 | 0 | 0 | 0 | 1 | 0 | 3 | 1 | 1 | 3 | 3 | 2 |
| S6 | 1 | 0 | 0 | 0 | 2 | 0 | 0 | 3 | 3 | 2 | 1 |
| S7 | 2 | 1 | 1 | 2 | 1 | 1 | 0 | 3 | 1 | 2 | 2 |
| S8 | 1 | 0 | 2 | 2 | 2 | 2 | 0 | 0 | 0 | 3 | 2 |
| S9 | 3 | 0 | 0 | 1 | 1 | 1 | 0 | 0 | 1 | 2 | 1 |
| S10 | 0 | 1 | 0 | 0 | 1 | 1 | 0 | 0 | 0 | 1 | 1 |
| S11 | 2 | 0 | 3 | 2 | 2 | 1 | 0 | 1 | 3 | 0 | 1 |
| S12 | 1 | 1 | 3 | 3 | 1 | 1 | 0 | 0 | 2 | 3 | 0 |
|  |  |  |  |  |  |  |  |  |  |  |  |
| Xg | G1 | G2 | G3 | G4 | G5 | G6 | G7 | G9 | G10 |  |  |
| G1 | 0 | 2 | 2 | 0 | 1 | 1 | 0 | 2 | 1 |  |  |
| G2 | 2 | 0 | 2 | 1 | 1 | 1 | 1 | 3 | 3 |  |  |
| G3 | 3 | 1 | 0 | 0 | 3 | 3 | 1 | 1 | 3 |  |  |
| G4 | 0 | 2 | 0 | 0 | 1 | 1 | 0 | 1 | 1 |  |  |
| G5 | 3 | 2 | 2 | 2 | 0 | 2 | 2 | 2 | 2 |  |  |
| G6 | 3 | 2 | 3 | 2 | 3 | 0 | 2 | 2 | 2 |  |  |
| G7 | 1 | 2 | 1 | 0 | 0 | 0 | 0 | 3 | 3 |  |  |
| G8 | 2 | 3 | 0 | 0 | 1 | 1 | 2 | 1 | 2 |  |  |
| G9 | 0 | 3 | 1 | 0 | 3 | 2 | 1 | 0 | 1 |  |  |
| G10 | 1 | 3 | 1 | 0 | 1 | 1 | 2 | 1 | 0 |  |  |

IV. Normalized direct-relaton matrix of economy and greenness enablers

| Ne | S1 | S2 | S3 | S4 | S5 | S6 | S7 | S8 | S9 | S10 | S11 | S12 |
| --- | --- | --- | --- | --- | --- | --- | --- | --- | --- | --- | --- | --- |
| SI | 0 | 0 | 0 | 0.158 | 0.105 | 0.105 | 0.158 | 0.105 | 0.105 | 0.053 | 0.053 | 0.053 |
| S2 | 0.053 | 0 | 0.158 | 0.158 | 0.053 | 0.053 | 0 | 0 | 0 | 0.105 | 0.105 | 0.105 |
| S3 | 0.053 | 0 | 0 | 0.158 | 0.053 | 0.053 | 0 | 0 | 0 | 0.053 | 0.105 | 0.053 |
| S4 | 0.158 | 0 | 0 | 0 | 0.053 | 0.105 | 0.053 | 0.053 | 0.105 | 0.053 | 0.105 | 0.053 |
| S5 | 0 | 0 | 0 | 0.053 | 0 | 0.158 | 0.053 | 0.105 | 0.053 | 0.158 | 0.158 | 0.105 |
| S6 | 0.053 | 0 | 0 | 0 | 0.105 | 0 | 0 | 0.053 | 0.158 | 0.158 | 0.105 | 0.053 |
| S7 | 0.105 | 0.053 | 0.053 | 0.105 | 0.053 | 0.053 | 0 | 0.158 | 0.158 | 0.053 | 0.105 | 0.105 |
| S8 | 0.053 | 0 | 0.105 | 0.105 | 0.105 | 0.105 | 0 | 0 | 0 | 0 | 0.158 | 0.105 |
| S9 | 0.158 | 0 | 0 | 0.053 | 0.053 | 0.053 | 0 | 0.105 | 0 | 0.053 | 0.105 | 0.053 |
| S10 | 0 | 0.053 | 0 | 0 | 0.053 | 0.053 | 0 | 0 | 0 | 0 | 0.053 | 0.053 |
| S11 | 0.105 | 0 | 0.158 | 0.105 | 0.105 | 0.053 | 0 | 0.105 | 0.053 | 0.158 | 0 | 0.053 |
| S12 | 0.053 | 0.053 | 0.158 | 0.158 | 0.053 | 0.053 | 0 | 0.053 | 0 | 0.105 | 0.158 | 0 |
|  |  |  |  |  |  |  |  |  |  |  |  |  |
| Ng | G1 | G2 | G3 | G4 | G5 | G6 | G7 | G8 | G9 | G10 |  |  |
| G1 | 0 | 0.095 | 0.095 | 0 | 0.048 | 0.048 | 0 | 0.143 | 0.095 | 0.048 |  |  |
| G2 | 0.095 | 0 | 0.095 | 0.048 | 0.048 | 0.048 | 0.048 | 0.143 | 0.143 | 0.143 |  |  |
| G3 | 0.143 | 0.048 | 0 | 0 | 0.143 | 0.143 | 0.048 | 0 | 0.048 | 0.143 |  |  |
| G4 | 0 | 0.095 | 0 | 0 | 0.048 | 0.048 | 0 | 0 | 0.048 | 0.048 |  |  |
| G5 | 0.143 | 0.095 | 0.095 | 0.095 | 0 | 0.095 | 0.095 | 0.095 | 0.095 | 0.095 |  |  |
| G6 | 0.143 | 0.095 | 0.143 | 0.095 | 0.143 | 0 | 0.095 | 0.095 | 0.095 | 0.095 |  |  |
| G7 | 0.048 | 0.095 | 0.048 | 0 | 0 | 0 | 0 | 0 | 0.143 | 0.143 |  |  |
| G8 | 0.095 | 0.143 | 0 | 0 | 0.048 | 0.048 | 0.095 | 0 | 0.048 | 0.095 |  |  |
| G9 | 0 | 0.143 | 0.048 | 0 | 0.143 | 0.095 | 0.048 | 0.048 | 0 | 0.048 |  |  |
| G10 | 0.048 | 0.143 | 0.048 | 0 | 0.048 | 0.048 | 0.095 | 0.048 | 0.048 | 0 |  |  |

V. Total-relation matrix of economy and greenness enablers

| Te | S1 | S2 | S3 | S4 | S5 | S6 | S7 | S8 | S9 | S10 | S11 | S12 |
| --- | --- | --- | --- | --- | --- | --- | --- | --- | --- | --- | --- | --- |
| SI | 0.236 | 0.043 | 0.156 | 0.402 | 0.333 | 0.349 | 0.234 | 0.328 | 0.302 | 0.323 | 0.385 | 0.265 |
| S2 | 0.230 | 0.034 | 0.281 | 0.366 | 0.235 | 0.247 | 0.068 | 0.158 | 0.144 | 0.326 | 0.358 | 0.260 |
| S3 | 0.184 | 0.023 | 0.090 | 0.296 | 0.186 | 0.195 | 0.054 | 0.124 | 0.115 | 0.216 | 0.283 | 0.166 |
| S4 | 0.335 | 0.033 | 0.130 | 0.218 | 0.251 | 0.303 | 0.130 | 0.240 | 0.264 | 0.281 | 0.364 | 0.221 |
| S5 | 0.188 | 0.041 | 0.152 | 0.264 | 0.207 | 0.352 | 0.107 | 0.277 | 0.206 | 0.395 | 0.427 | 0.276 |
| S6 | 0.198 | 0.031 | 0.110 | 0.172 | 0.260 | 0.170 | 0.054 | 0.199 | 0.263 | 0.342 | 0.321 | 0.193 |
| S7 | 0.351 | 0.093 | 0.236 | 0.396 | 0.308 | 0.319 | 0.092 | 0.381 | 0.342 | 0.343 | 0.458 | 0.326 |
| S8 | 0.231 | 0.030 | 0.239 | 0.319 | 0.293 | 0.302 | 0.069 | 0.173 | 0.154 | 0.244 | 0.415 | 0.265 |
| S9 | 0.304 | 0.027 | 0.118 | 0.243 | 0.225 | 0.232 | 0.073 | 0.257 | 0.135 | 0.247 | 0.329 | 0.199 |
| S10 | 0.060 | 0.065 | 0.059 | 0.080 | 0.119 | 0.121 | 0.020 | 0.060 | 0.051 | 0.094 | 0.148 | 0.113 |
| S11 | 0.287 | 0.037 | 0.280 | 0.333 | 0.306 | 0.275 | 0.079 | 0.277 | 0.205 | 0.387 | 0.292 | 0.235 |
| S12 | 0.243 | 0.084 | 0.292 | 0.380 | 0.252 | 0.262 | 0.072 | 0.220 | 0.154 | 0.341 | 0.422 | 0.176 |
|  |  |  |  |  |  |  |  |  |  |  |  |  |
| Tg | G1 | G2 | G3 | G4 | G5 | G6 | G7 | G8 | G9 | G10 |  |  |
| G1 | 0.135 | 0.255 | 0.195 | 0.043 | 0.170 | 0.154 | 0.104 | 0.249 | 0.222 | 0.179 |  |  |
| G2 | 0.252 | 0.222 | 0.222 | 0.095 | 0.203 | 0.182 | 0.174 | 0.276 | 0.302 | 0.296 |  |  |
| G3 | 0.305 | 0.256 | 0.152 | 0.065 | 0.286 | 0.265 | 0.174 | 0.157 | 0.223 | 0.300 |  |  |
| G4 | 0.070 | 0.176 | 0.062 | 0.028 | 0.109 | 0.100 | 0.054 | 0.066 | 0.120 | 0.114 |  |  |
| G5 | 0.316 | 0.330 | 0.245 | 0.150 | 0.174 | 0.238 | 0.227 | 0.254 | 0.288 | 0.282 |  |  |
| G6 | 0.343 | 0.355 | 0.306 | 0.159 | 0.324 | 0.173 | 0.244 | 0.272 | 0.311 | 0.307 |  |  |
| G7 | 0.136 | 0.220 | 0.128 | 0.028 | 0.098 | 0.084 | 0.075 | 0.090 | 0.238 | 0.226 |  |  |
| G8 | 0.211 | 0.292 | 0.110 | 0.041 | 0.152 | 0.138 | 0.183 | 0.119 | 0.187 | 0.221 |  |  |
| G9 | 0.153 | 0.305 | 0.169 | 0.058 | 0.260 | 0.201 | 0.157 | 0.174 | 0.153 | 0.201 |  |  |
| G10 | 0.165 | 0.280 | 0.147 | 0.041 | 0.150 | 0.137 | 0.178 | 0.153 | 0.179 | 0.130 |  |  |
