# Supplementary material for: A duo-theme cloud model DEMATEL approach for exploring the cause factors of green supply chain management
Source: PLoS One. 2024 Mar 28;19(3):e0294684. doi: 10.1371/journal.pone.0294684 (PMC10977737; doi:10.1371/journal.pone.0294684)
Supplement: Attachment — Submitted filename: Response to Reviewer #2.docx [file pone.0294684.s003.docx]

Dear reviewer #2,

Thank you very much for your positive and constructive comments and suggestions, we have studied your comments carefully and have revised the manuscript. The amendments are highlighted in blue in the revised manuscript.

Following are the responds for your comments:

**Response:**

(1) The explanation has been enhanced to illustrate the research object and describe specific cases.
(2) Explanations have been enhanced regarding what criteria the experts select.
(3) An enhanced explanation has been provided to illustrate the definitions of both.
(4) The data of the calculation process has been moved to the appendix; the relevant method description has been enhanced.

**Note: All revisions are indicated in blue font**.
